# A Review of Foreign Language Enjoyment and Engagement

**DOI:** 10.3389/fpsyg.2021.737613

**Published:** 2021-08-12

**Authors:** Yijun Zeng

**Affiliations:** Department of International Business, Hainan College of Foreign Studies, Wenchang, China

**Keywords:** positive psychology, motivation, engagement, foreign language enjoyment, second language acquisition, anxiety

## Abstract

The introduction of positive psychology into foreign/second language learning has led to a multitude of novel theoretical and empirical studies. Foreign language enjoyment (FLE) is regarded as a response to the widely examined concept of classroom anxiety. The majority of these studies have investigated the effect of learners’ and teachers’ characteristics (Xie and Derakhshan, 2021) pertaining to FLE on learners’ academic achievement and their engagement in classroom tasks. Following a seminal study by Dewaele and MacIntyre (2014) and the development of the primary FLE scale, some researchers evaluated the extent of learners’ enjoyment in the language learning environment; these studies approved the effectiveness and prominence of FLE throughout the learning process. The present review is an attempt to review studies on FLE during the past two decades. The related literature confirms the significance and efficiency of promoting FLE in the classroom because it brings about higher levels of motivation and engagement among language learners and leads to prolonged success and achievement. A summary of the major efforts regarding this area of research is presented in this study.

## Introduction

This manuscript is an attempt to investigate foreign language enjoyment (FLE), as a prominent feature of positive education (PE). The introduction of Positive Psychology (PP) can contribute to leading people toward success in their lives (Seligman and Csikszentmihalyi, 2000; Csikszentmihalyi and Nakamura, 2011). MacIntyre and Gregersen (2012) claimed that PP should be implemented in SLA because it is necessary to examine and affect students’ emotions so that they can flourish and move toward their desirable objectives in future. Since 2016, there has been a shift toward PP in SLA (Lake, 2013). The proponents of this novel area of inquiry have intended to prepare the grounds for further research on the impact of PP in education and particularly language teaching and learning (Dewaele and MacIntyre, 2014; Gregersen and MacIntyre, 2017; Fathi and Derakhshan, 2019; Derakhshan et al., 2021; Pishghadam et al., 2021). This study attempts to appraise the present literature on FLE to observe the rationale and purposes of the studies and their empirical findings, determine those areas of research that have been disregarded, and propose some new topics for further research.

## Theoretical Background

### FLE as a PP Construct

Damasio (1994) and Fredrickson (2004) argued that learning is beyond affective factors and involves a multitude of factors such as communication, rapport, and identity. Having focused on emotion theory and earlier studies on affective factors in FL/SL learning, Seligman and Csikszentmihalyi (2000) introduced positive concepts into educational psychology. They asserted that it is necessary for language teaching/learning practitioners to consider well-being, hope, empathy, mindfulness, communicative skills, etc., to make a balance in the literature (Snyder and Lopez, 2009; Macintyre et al., 2019).

Seligman (2018) identified the building blocks of well-being in his PERMA model (Positive emotion, Engagement, Relationships, Meaning, and Achievement). Accordingly, FLE can be regarded as the realization of the Positive Emotion element in this model. Moreover, Fredrickson (2001) highlighted the “broadening-and-building” nature of positive emotions as the psychological capacity of individuals, which can lead to expansion of their perception. In language learning, FLE refers to learners’ endeavors to meet learning challenges and broaden their knowledge and proficiency in the classroom (MacIntyre, 2016). Moreover, Botes et al. (2020) concluded that FLE occurs when learners can find appropriate responses to their psychological needs in the classroom. Pekrun et al. (2007) highlighted that enjoyment can result in persistent determination as well as positive and enthusiastic engagement in educational tasks (Mierzwa, 2018). Furthermore, language learners’ evaluation of their own behavior may lead to the feeling of anxiety or foreign language enjoyment (Wei et al., 2019). Consequently, the implementation of PP in language learning might result in academic attainment and success.

## Empirical Studies

Empirical studies on FLE can be categorized into the following four types of research:

### Validity of the FLE Measurements

**FIGURE A1:**
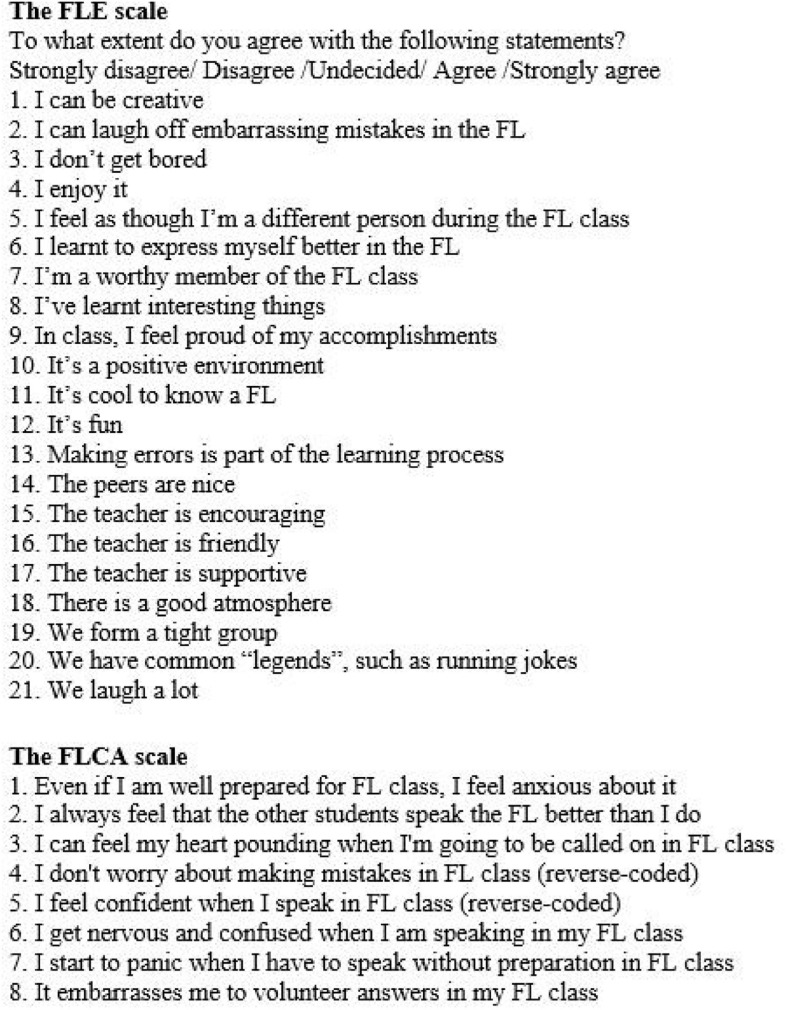
FLE and FLCA scales proposed by Dewaele and MacIntyre (2014).

Since the study by Dewaele and MacIntyre (2014) is regarded as the building block of further research in FLE, it is imperative to start this section by highlighting the procedure of this research. They aimed to explore the correlation between enjoyment and anxiety in the language learning process. For this purpose, 1746 language learners with 90 different linguistic backgrounds and nationalities were selected. These learners were studying English, French, Spanish, Dutch, and German. The participants were selected to complete a researcher-made questionnaire on the web. The instrument contained 29 items (21 items developed to measure FLE observing positive emotions regarding the teacher, peers, and the learning experience, as well as eight items were extracted from the FLCA Scale) (see Appendix Figure A1). Once the questionnaire was completed, participants were asked to answer the following open-ended question: “Describe one specific event or episode in your FL class that you really enjoyed, and describe your feeling in as much detail as possible” (Dewaele and MacIntyre, 2014, p. 246). Dewaele and MacIntyre (2016) applied some modifications to the original FLE Scale and developed a 14-item questionnaire with an internal consistency coefficient of 0.86. Given the acceptable reliability and validity of the proposed instrument, many scholars have employed this FLE scale or the translated versions in their studies (e.g., the Chinese version developed by Li et al., 2018).

Li et al. (2018), used a mixed-methods approach to evaluate the validity of the Chinese version of the FLE scale. Eventually, they proposed a 11-item scale within three factors of FLE-Private, FLE-Teacher, and FLE-Atmosphere. In a similar vein, Jin and Zhang (2018) investigated the relationship between FLE and academic achievement among 320 Chinese students. They developed a 17-item scale [English Classroom Enjoyment Scale (ECES)] examining three factors including Enjoyment of Teacher Support, Enjoyment of Student Support, and Enjoyment of Foreign Language Learning, which was revised by the same authors in 2019. The revised version of ECES contained 16 items and was claimed to provide a more reliable instrument with more efficient psychometric properties.

Alongside the developments in the measurement of FLE in the above mentioned studies, Elahi Shirvan et al. (2021) employed a longitudinal confirmatory factor analysis to evaluate the psychometric properties of FLE scales over time. They also concluded that language learners with higher levels of L2 FLE did not experience great changes over time, while those with the initially lower level of FLE reported significant changes in this construct in the long run.

### The Association Between FLE and Demographic Variables

Researchers have examined the relationship between different demographic variables and FLE. In terms of age, for instance, Dewaele et al. (2018) as well as Dewaele and MacIntyre (2014) asserted that younger language learners indicated lower levels of FLE. They also revealed that university students showed higher levels of FLE compared to high-school students. Besides, there has been inconsistencies among scholars over the impact of gender on FLE. Some studies provide that female language learners have demonstrated greater FLE (Dewaele and MacIntyre, 2016), whereas other researchers argued that there is an insignificant difference between the two gender in term of language enjoyment (Mierzwa, 2018; Alenezi, 2020). Since there has been a few researches on the impact of demographic characteristics on FLE, it is imperative to conduct further studies to provide reliable outcomes and implications.

### The Link Between FLE and Individual Difference Variables

Foreign language enjoyment has been widely evaluated in association with foreign language anxiety (FLA). Dewaele and MacIntyre (2014) are considered the Pioneering authors in this regard as they have attempted to observe and compare negative (FLA) and positive (FLE) emotions. Other scholars have also examined the correlation between FLA and FLE; the summary of findings of such studies highlights a fairly negative relationship between these two concepts (Dewaele and Dewaele, 2017; Dewaele and Alfawzan, 2018; Resnik and Dewaele, 2020).

On the other hand, research on FLA has focused on different variables such as academic achievement, willingness to communicate (WTC), and etc. Since WTC refers to the learner’s intention to speak up and communicate with the teacher and peers in the classroom, there might be a correlation between WTC and learners’ FLE (Khajavy et al., 2018). Correspondingly, scholars have decided to conduct similar studies concerning FLE in the classroom (Dewaele, 2019; Elahi Shirvan and Taherian, 2020). Consequently, Dewaele (2019) argued that it is necessary for language teachers to create a positive atmosphere in the classroom, which can help develop and promote WTC. As a result of such positive environment, language learners could also expand their language learning enjoyment so as to succeed in achieving their linguistic-related objective.

### FLE as a Complex and Dynamic Construct

Several studies demonstrated that FLE is a complicated and dynamic concept. Dewaele and Dewaele (2017) implemented a dynamic approach to investigate changes in FLE. All the participants in their study reported a growing FLE over time. They further asserted that learner’s variables might not be able to predict language learning achievement, which indicated the dynamic nature of FLE. In addition, Elahi Shirvan and Talebzadeh (2018) argued that language enjoyment changes from topic to topic and among individuals, since learners have experienced various degrees of FLE throughout the learning process in the classroom. This finding can contribute to the effectiveness of interpersonal as well as inherent variables on language learners experiences.

Similarly, Li et al. (2018) conducted a study on the fluctuation of enjoyment and anxiety in the classroom. They further argued that poor linguistic capabilities and the lack of motivation can lead to various levels of FLE and FLCA among language learners. Moreover, Elahi Shirvan et al. (2020) aimed to investigate the extent of changes in FLE among language learners. For this purpose, they employed an ecological momentary evaluation approach using journals, interviews, and enjoyments. Findings of their research indicated that FLE changes from moment to moment and also from months to months.

Additionally, a seminal study was carried out by Dewaele and Dewaele (2020) so as to assess the effect of two different teachers on probable changes in FLE among language learners. They concluded that the teacher’s use of target language in the classroom and learners’ positive perception of the teacher can lead to a greater level of FLE.

## Suggestions for Future Research

Dewaele and MacIntyre (2016) concluded that foreign language enjoyment is primarily influenced by the teachers’ behavior. Consequently, Li et al. (2018) declared that teachers can influence and improve language learners’ motivation and engagement in classroom tasks through allowing more foreign language input in the classroom by encouraging student-student interactions. Moreover, there is limited research in terms of the relationship between FLE/Second Language Enjoyment (SLE) and different language skills. Since enjoyment is positively associated with language learners’ proficiency, it is recommended to conduct further research to investigate the role of FLE in the development and improvement of language skills. It is noteworthy that since positive psychology constructs are dynamic and complicated, Dörnyei and Ryan (2015) proposed the necessity to move toward dynamic research approaches that contribute to the changing nature of language learning concepts in academic studies; for this purpose, future research on anxiety and enjoyment should consider longitudinal perspectives (Elahi Shirvan and Taherian, 2021).

## Author Contributions

The author confirms being the sole contributor of this work and has approved it for publication.

## Conflict of Interest

The author declares that the research was conducted in the absence of any commercial or financial relationships that could be construed as a potential conflict of interest.

## Publisher’s Note

All claims expressed in this article are solely those of the authors and do not necessarily represent those of their affiliated organizations, or those of the publisher, the editors and the reviewers. Any product that may be evaluated in this article, or claim that may be made by its manufacturer, is not guaranteed or endorsed by the publisher.
